# New Challenges in the Diagnosis and Treatment of Primary Cutaneous Aspergillosis in Extensive Pediatric Burns

**DOI:** 10.3390/jof11040281

**Published:** 2025-04-02

**Authors:** Doina Iulia Nacea, Dan Mircea Enescu, Raluca Tatar, Olguta Anca Orzan, Laura Sorina Diaconu

**Affiliations:** 1 Department of Plastic Reconstructive Surgery, “Grigore Alexandrescu” Clinical Emergency Hospital for Children, “Carol Davila” University of Medicine and Pharmacy, 020021 Bucharest, Romania; iulia.nacea@umfcd.ro (D.I.N.);; 2 Department of Plastic Reconstructive Surgery and Burns, “Grigore Alexandrescu” Clinical Emergency Hospital for Children, 010621 Bucharest, Romania; 3 Department of Oncologic Dermatology, “Elias” Emergency University Hospital, “Carol Davila” University of Medicine and Pharmacy, 020021 Bucharest, Romania; 4 Clinic of Dermatology, “Elias” Emergency University Hospital, 011461 Bucharest, Romania; 5 Department of Internal Medicine III and Gastroenterology, ”Carol Davila” University of Medicine and Pharmacy, 020021 Bucharest, Romania; sorina.diaconu@umfcd.ro; 6 Emergency University Hospital of Bucharest, 050098 Bucharest, Romania

**Keywords:** primary cutaneous aspergillosis, extensive burns, pediatric burns, Meek micrografting, skin grafts, topical treatment, systemic treatment, fungal infection

## Abstract

The aim of this study is to share our experience regarding the diagnosis and therapeutic management of primary cutaneous aspergillosis (PCA) in the burn patient, an uncommon infection associated with increased mortality, morbidity, and treatment costs. The uniqueness of this article is the presence of PCA in pediatric patients where the Meek micrografting technique was used. We performed a retrospective study from June 2020 to November 2024. The inclusion criteria were the concomitant presence of burn injuries and confirmed PCA. We identified six patients, aged between 12 and 17 years, admitted with deep burns ranging from 55% to 90% of the total body surface area (TBSA). They required complex ICU treatment and underwent extensive excision–grafting surgeries. The suspicion of infection was raised by changes in the appearance of wounds. Systemic and topical antifungal treatment was established in patients after a PCA diagnosis. Five out of the six cases had a favorable outcome. The use of the Meek micrografting technique in burn treatment represents a new challenge in the treatment of PCA due to the polyamide gauze that covers the micrografts. Early detection and appropriate topical antifungal agents combined with systemic treatment may save the infected grafts and limit the infection spread without necessarily removing the polyamide gauze.

## 1. Introduction

Burns are a significant problem for all healthcare systems due to the increased risk of death, long hospitalization periods burdened with multiple complications, increased treatment costs, the need for highly specialized medical personnel, and the necessity of developing burn centers aligned with international norms [1]. In the last decade, there has been a noticeable increase in survival rates among patients with extensive and massive burns through the construction of new burn centers, a decrease in transfer time to specialized units, the improvement of intensive care treatment and antibiotic therapy, and the development of new options for covering large skin defects that allow for the early excision of extensive cutaneous [2,3,4]. The Meek micrografting technique is one of these new covering solutions, frequently used in our hospital, which allows for the excision of extensive burns even in the absence of allografts and other dermal substitutes. This technique, first proposed by Cicero Parker Meek in 1958, was revisited and modified by Dutch specialists in 1990. The first step involves harvesting split-thickness skin grafts, as in any autografting surgery. Subsequently, using special Meek cutting devices, the autografts are divided into small squares; then, an adhesive spray is used, and the micrografts are attached to a pre-folded polyamide gauze. Unfolding this gauze provides an expansion rate of up to 1:9, allowing for extensive burn wound excision and coverage. The gauze may stay in place for 10–14 days, sometimes longer. After its removal, the graft take is assessed [5,6].

This decrease in mortality has proven to be associated with a rise in the incidence of very rare nosocomial fungal infections. In total, 50 to 75% of nosocomial fungal infections are caused by *Candida albicans*, followed by other candida species (spp.) and *Aspergillus* spp. [3]. Primary cutaneous aspergillosis (PCA) is an invasive fungal infection rarely encountered in medical practice, even in dedicated burn centers. It appears due to direct fungus inoculation in preexisting skin lesions [7]. When *Aspergillus* disseminates from a distant site, most frequently from the lungs to the skin, secondary cutaneous aspergillosis (SCA) appears, which is a more common form than PCA [8,9]. Only a few articles dedicated to the management of burn patients also mention fungal infections and PCA, most of them being single-case reports [10,11,12] or literature reviews [5,13,14].

Therefore, we consider it important to share the experience of the Burn Unit at the “Grigore Alexandrescu” Clinical Emergency Hospital for Children, Bucharest, Romania, regarding the diagnosis and therapeutic management of PCA in patients with extensive burns, an uncommon infection associated with increased mortality, morbidity, and treatment costs [15,16]. As far as we know, there are no other publications reporting its occurrence in patients in whom the Meek micrografting technique was used. At the same time, this is one of the largest patient samples with PCA in extensive pediatric burns.

## 2. Materials and Methods

We performed a retrospective study, analyzing the files of all burn patients admitted to our department. The inclusion criteria were the presence of a burn injury concomitantly with confirmed cutaneous aspergillosis. The investigation period ran from June 2020 (the date of the first positive wound culture for *Aspergillus* spp. in a burn patient in our department) to November 2024. The diagnosis of cutaneous aspergillosis was based on positive mycological analyses and was firmly established by the proven invasion of healthy tissues by histopathological analyses of punch biopsy specimens. A lack of tissue invasion confirmed by histopathological analyses represents the exclusion criterion and classifies the cases as colonization. The species of *Aspergillus* were established by our hospital’s microbiologists, depending on the appearance of the colonies and microscopic examinations. For antifungal susceptibility, RPMI medium was used with the E-Test method.

The study was approved by the Ethics Committee of the Hospital (Approval no. 21/17141/6 June 2024).

From the medical files of the selected cases, we collected demographic data regarding the patients (age and gender), clinical descriptions of the burn injuries (etiology, depth, surface, and affected sites), the surgical approach, the timing of PCA identification, the local and systemic treatment decided on for each patient in relation to this condition and their further subsequent outcomes.

## 3. Results

During the investigation period, our department admitted and treated a total of 1109 pediatric burn patients. Among these, 42 cases with extensive and massive burns (over 50% of the TBSA, predominantly with third-degree burns) were surgically excised and covered using the Meek micrografting technique. The rest of the burns were either less extensive or more superficial. Within the whole group, we retrieved seven cases of burn patients who had positive mycological analyses for *Aspergillus* spp. during hospitalization. In one of the cases, the histopathological analyses revealed a lack of *Aspergillus* sp. invasion in healthy tissue, and he was, therefore, excluded from the study. No other molds were identified in mycological analyses.

Other sites of infection were ruled out by imaging investigations. At the moment of the PCA diagnosis, all the selected patients had antifungal prophylaxis with intravenous fluconazole for Candida in their treatments. From that point, we switched from fluconazole to voriconazole, initiated with an intravenous infusion loading dose of 6 mg/kg every 12 h for the first 24 h, followed by a maintenance dose of 3 mg/kg every 12 h. Intravenous voriconazole was continued after mycological analyses were negative. It was stopped after it was decided to put an end to the antibiotic therapy, and the central venous catheter was removed.

### 3.1. Case Presentations

#### 3.1.1. Case 1

**Figure 1:**
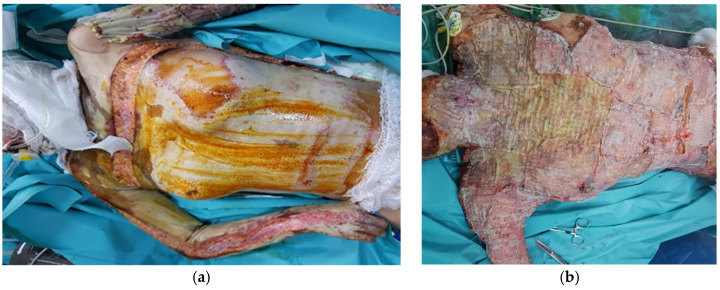

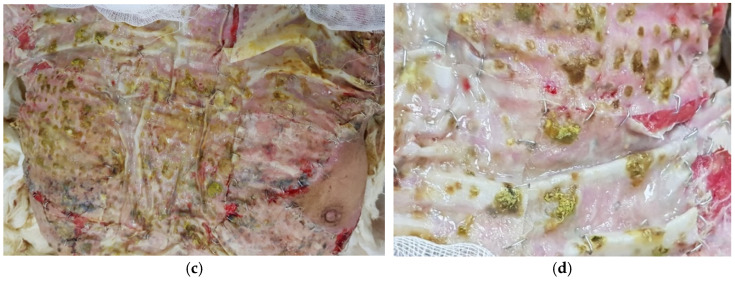
(**a**) Anterior trunk before the second surgery, on the 10th day of hospitalization. (**b**) Anterior trunk after excision and covering with the Meek micrografting technique. (**c**,**d**) Anterior thorax after development of yellowish-green mycelia colony-like formations suggestive of *Aspergillus flavus*.

A 17-year-old girl, a victim of fire assault, was referred to our clinic from another hospital, 21 h after the aggression. The patient had 90%-total body surface area (TBSA) third-degree burns, airway burns, and an Abbreviated Burn Severity Index Score (ABSI score) of 13. She required emergency escharotomies on the neck, trunk, and both the upper and lower limbs (Figure 1a). From the medical history, we noted appendicectomy and oligomenorrhea. She remained in the ICU for the first 15 days. On the fourth day of hospitalization, the left upper limb and the left lower limb burn wounds were excised and grafted. During the second surgery, which took place a week later, the anterior trunk, anterior neck, and right upper limb were excised and grafted (Figure 1b). The patient was extubated 14 days postburn. In the next two surgeries, the burn eschars located on the right lower limb and on the posterior trunk were excised. The Meek micrografting technique was used for coverage in all four surgeries with expansion rates of 1:4, 1:6, and 1:9.

Two days after the fourth surgery, there was a change in the appearance of the dressings on the upper limbs and anterior thorax. Yellowish-green colony-like formations developed on these sites (Figure 1c,d). Mycological analyses revealed *Aspergillus flavus*. No bacteria grew in the culture. Histopathological analyses proved the presence of fungal elements in viable tissue. The local evolution of the infection was widespread, identifying new colonies of *Aspergillus* in the posterior trunk and thighs. Antifungal susceptibility testing was possible only in an external laboratory and showed susceptibility to voriconazole (minimum inhibitory concentration (MIC) = 0.08 μg/mL) and itraconazole (MIC = 0.12 μg/mL) and resistance to amphotericin B (MIC = 1.63 μg/mL). Systemic fluconazole treatment (provided as prophylactic therapy in the context of long-term antibiotic administration) was changed to voriconazole. The polyamide gauze that covered the micrografts was removed from the anterior trunk and both upper limbs. Topical treatment of aspergillosis consisted of the application of voriconazole and 1% silver sulfadiazine cream, alternatively. *Aspergillus* colonies from the posterior trunk and lower limbs, where the polyamide gauze stayed in place, disappeared and, the micrografts grew and merged. The areas where the polyamide gauze was removed developed granulation tissue that required another skin graft. The patient was discharged after 5 months of hospitalization. She was followed up 4 years after the accident with no recurrence of PCA.

#### 3.1.2. Case 2

A 13-year-old boy was admitted with 80%-TBSA third-degree flame burns and mechanically ventilated. He required emergency escharotomies on the trunk and both the upper and lower limbs. The first surgery took place on the seventh day, when the trunk, the right upper limb, and the left thigh were excised and grafted. On the 14th day of hospitalization, he benefited from excision–grafting of the lower-limb burns. On the 21st day after the accident, the remaining full-thickness burns (neck, face, right hand, and external genitalia) were excised and grafted. In all surgeries, coverage was achieved using the Meek micrografting technique. Two weeks after the third surgery, there was a change in the appearance of the dressings on the anterior trunk. Mycological analyses revealed the presence of *Aspergillus* spp. Antifungal susceptibility testing was not possible. For the topical treatment of aspergillosis, we used voriconazole, this time without removing any of the polyamide gauze. Systemic fluconazole treatment was also changed to voriconazole. A negative wound culture was achieved after 2 weeks. Micrografts grew and merged. The boy was discharged after 80 days of hospitalization.

#### 3.1.3. Case 3

**Figure 2:**
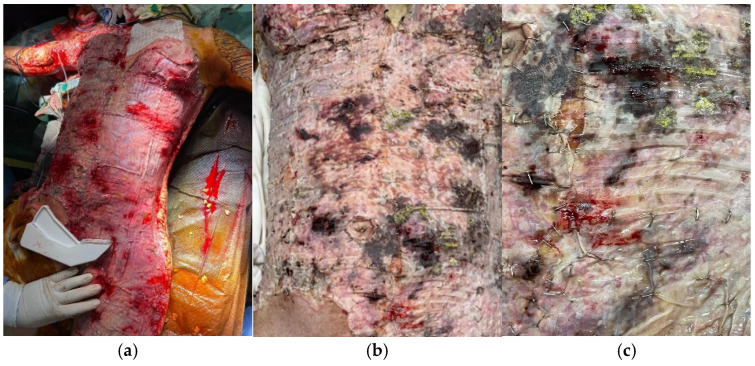
(**a**) Anterior trunk at the end of the excision grafting surgery. (**b**,**c**) Anterior trunk on the 16th day of hospitalization when yellowish-green colony-like formations of *Aspergillus flavus* became visible.

A 14-year-old girl was admitted with 85%-TBSA third-degree flame burns from a home accident. She was in critical condition from the beginning, with hemodynamic and respiratory instability, severe coagulation disorders, thrombocytopenia, and renal failure for which hemodiafiltration was performed. The patient required emergency escharotomies of the trunk and both upper limbs. On the 12th day postburn, we excised eschars from the anterior trunk, both upper limbs, and the anterior surface of the left thigh, about 30% of the TBSA. The wounds were covered with Meek micrografts (Figure 2a). Postoperatively, hemodiafiltration was resumed. Changes in the dressings and wound appearance were noticed on the 4th day after the surgery and the 16th after admission (Figure 2b,c). Mycological analyses revealed *Aspergillus flavus*, and histopathological analyses established its invasive character. The general state of the patient worsened owing to multiple system organ failure, and we registered her death 20 days after the accident. All blood cultures collected during hospitalization were negative.

#### 3.1.4. Case 4

**Figure 3:**
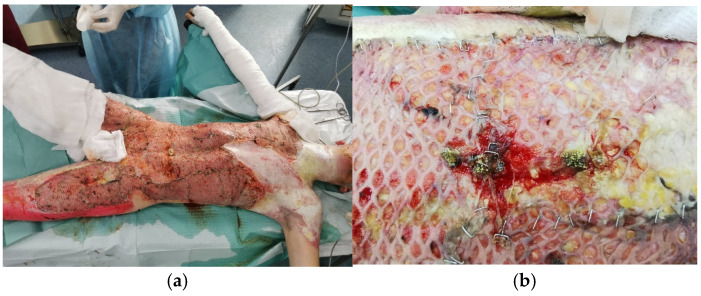
(**a**) Patient’s appearance 2 days after excision–grafting surgery. For coverings, we used meshed grafts (**b**) left tight a week after excision–grafting. Yellow mycelia colony-like formations evocative of *A. flavus* can be observed.

A 17-year-old boy was transferred from another hospital with 55% TBSA third-degree electrical burns. Escharotomy of the trunk, both thighs, and external genitalia was necessary upon admission. Fourteen days after the accident, we excised 25% of the TBSA and covered the wound using meshed autografts (Figure 3a). A week after the surgery, we noticed the development of some yellow colonies in his left thigh grafts. Mycological analyses revealed the presence of *Aspergillus flavus* (Figure 3b). Voriconazole was used for topical and systemic treatment of this local infection. The local evolution was favorable, with the disappearance of Aspergillus colonies. No grafts were lost. The patient required two more minor excision–grafting surgeries. He was discharged after 45 days of hospitalization.

#### 3.1.5. Case 5

**Figure 4:**
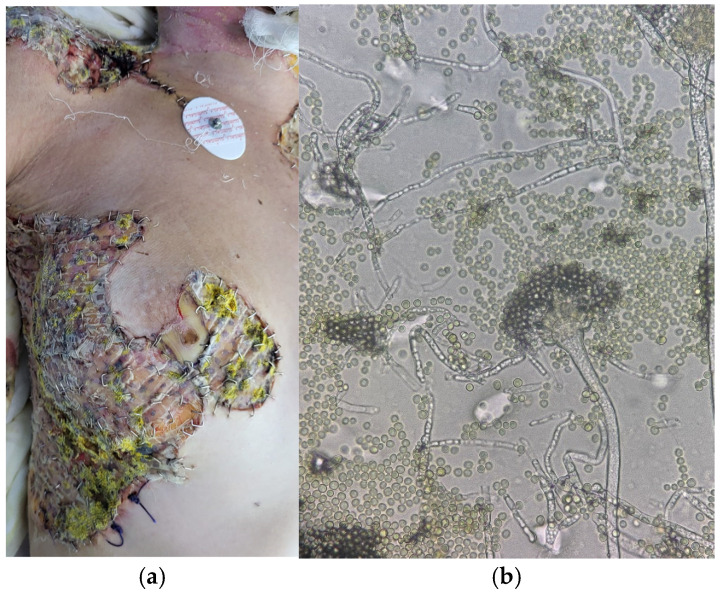
(**a**) Yellow mycelia colony-like formations suggestive of Aspergillus-like molds developed on the polyamide gauze covering the micrografts. (**b**) Microscopy picture under 40× magnification showing hemispherical vesicles and conidiophores of *Aspergillus flavus*.

A 15-year-old girl was referred to our department with 75%-TBSA deep-partial and full-thickness burns produced by a high-tension arc flash after climbing onto a train. The accident occurred 24 h before she was transferred by plane from another medical facility located 600 km away from us. In the emergency unit, the patient suffered cardiorespiratory arrest that responded to resuscitation maneuvers. She required emergency escharotomies on the left upper limb. She remained in the ICU for the first 12 days. On the seventh day postburn, the full-thickness burns from the anterior trunk, both arms, and the cervical area were excised and grafted using the Meek micrografting technique. Two weeks later, a second excision–grafting surgery was performed. After the second surgery, yellow mycelia colony-like formations developed on the polyamide gauze of the anterior trunk (Figure 4a). The microscopy examination described spherical vesicles covered with biseriate philaids radiating from all sides, suggestive of *Aspergillus flavus* (Figure 4b). Antifungal sensibility testing was not possible. Using topical voriconazole alternatively with silver sulfadiazine and systemic voriconazole, the colonies disappeared in less than a week with negative mycological analyses.

The patient required four more surgeries, two to cover granulation tissue areas and two for bedsores. She was released after 2 months of hospitalization with negative mycological analyses. After discharge, she benefited from monthly medical checks during which no recurrences of PCA were identified.

#### 3.1.6. Case 6

A 12-year-old boy was admitted for an 80%-TBSA deep-partial (20% TBSA) and full-thickness (60% TBSA) high-voltage electrical injury after climbing onto a train, with associated fall trauma with a first-rib fracture. Emergency escharotomies were necessary on both the arms and trunk. After 6 days of hospitalization, we excised burn eschars accounting for 20% TBSA and the defect was covered with meshed grafts. A week later, the other 16% of the TBSA was excised and grafted. This time, because of a lack of donor sites for autografts, the Meek micrografting technique was used to cover the defects. On the 17th day of treatment, green, filamentous colonies were noticed on the anterior trunk. A culture and microscopical examination revealed two types of *Aspergillus* spp., *Aspergillus flavus* and *Aspergillus niger.* This is the only case of a burned patient from our hospital who developed cutaneous aspergillosis caused by two species of *Aspergillus* at the same time. For antifungal susceptibility, plates with cultures were sent to an external laboratory because such tests could not be performed at our hospital at that moment. *Aspergillus flavus* proved to be sensitive to posaconazole (MIC = 0.25 μg/mL) and voriconazole (MIC = 0.25 μg/mL), and *Aspergillus niger* was resistant to amphotericin B (MIC = 2.25 μg/mL) and sensitive to posaconazole (MIC = 0.016 μg/mL) and voriconazole (MIC = 0.006 μg/mL). Topical and systemic treatment with voriconazole led to the disappearance of macroscopic fungal colony-like formations in less than 5 days, without losing any grafts. On the 20th day of hospitalization, necroses on the posterior trunk and the right thigh were excised (about 18% of the TBSA). The Meek micrografting technique was used for coverage.

After one month of hospitalization, without any previous suggestive symptoms, the patient developed a gastrointestinal hemorrhage that was endoscopically explored and treated by a pediatric surgeon. He was subsequently transferred to the ICU. This complication had no impact on his local infection status. We did not register other *Aspergillus* sp. colonies on the grafted areas. Following his recovery from the bleeding episode, he underwent several skin-grafting surgeries for the remaining areas of granulation tissue. The patient was discharged after 73 days.

## 4. Discussion

PCA appears through the direct inoculation of the fungus in a disruption of skin integrity, usually in immunocompromised patients. The burn patient presents two predisposing conditions at the same time: extensive cutaneous lesions and immunosuppression with reduced neutrophil and T-cell function [17]. The risk of PCA appearance is correlated with the extensiveness of the burn injury (% of the TBSA), the duration of wound coverage, the exposure to broad-spectrum antibiotics and glucocorticoid therapy, concomitant degenerative disease, and the increased use of central IV catheters [2,16,18]. In our series, all the patients were teenagers, aged between 12 and 17 years, without any known health issues, and hospitalized for extensive deep burns ranging between 55 and 90% of the TBSA (average TBSA: 77.5%). The extensiveness of primary skin lesions is directly correlated with the development of local fungal infections, as described by multiple publications [2,19,20]. Fournier et al. reported PCA occurrence in three patients aged between 17 and 51 years old admitted with burns covering between 64 and 81% of the TBSA [15]. Anh-Tram Que et al. found PCA in a 61-year-old male admitted to Vietnam NIB with burns estimated to cover 50% of the TBSA [11], while Aries et al. described the occurrence of PCA in a 53-year-old woman admitted for full-thickness burns estimated to cover 60% of the TBSA [12].

In all identified patients, colonies of *Aspergillus* appeared on excised and grafted areas between the 16th and 28th days after admission. Sharma et al. reported similar results, with the maximum incidence of burn wound fungal infection being registered during the third and fourth weeks of hospitalization [21]. Our case series supports this clinical finding.

Usually, the clinical aspect of PCA is not specific and is described as macules, papules, firm or fluctuant erythematosus nodules, hemorrhagic lesions, or necrotic areas in patients with primary or acquired immunodeficiency [7,19,20,21]. In the absence of adequate treatment, the evolution is toward the lysis of the grafts and spreads to other organs and systems. The macroscopic appearance of all our patients was gross yellowish-green fungal colony-like formations, similar to those grown on culture media in the laboratory. The extent of the fungal colonies on the surface of the grafts and their particular aspects contributed to establishing the diagnosis in a shorter time than in other locations. From the studied literature, we identified this description in only one case report article [11]. This aspect could be explained by the presence of exudate in the recently grafted wound; the polyamide gauze used to fix the micrografts; and the wet dressings used to cover the grafts, which favor the maintenance of a moist environment, rich in proteins, creating favorable conditions for *Aspergillus* sp. multiplication not only in soft tissues but also on their surfaces. In five out of the six presented cases, due to the limited autograft donor sites required for covering the defects after the excisions of full-thickness burns, the Meek micrografting technique was used. This technique is a reliable and versatile method for covering extensive burn areas with minimal donor sites. We have been using it in our burn unit for over 6 years, and it has remarkably improved the outcomes of severely burned patients, considering the fact that there are no allografts or other skin substitutes available.

The diagnosis of cutaneous aspergillosis requires positive cultures associated with histological evidence of invasion in viable tissue by biopsy [15,22]. For cultures, the Sabouraud Dextrose Agar medium was used, which is the most common type of medium used for the isolation, growth, and maintenance of species of fungi and yeasts. Molds were identified based on their macroscopic and microscopic morphological characteristics.

Histopathological analyses highlight the presence of mold elements in healthy tissue. Usually, the skin biopsy specimen contains dermis and subcutaneous fat. All our patients underwent fascial excision before PCA development, which explains the lack of the above-mentioned structures in the specimen and the presence of granulation tissue.

Although *Aspergillus* spp. are ubiquitous fungi with over 300 described species, only a few are pathogenic to humans. Most cases of PCA are caused by *Aspergillus flavus* complex and *A. fumigatus* complex, followed by *Aspergillus niger* complex, *Aspergillus terreus* complex, and *Aspergillus ustus* complex [9,23,24,25,26]. In our series, *Aspergillus flavus* complex was responsible for PCA in four cases; in one patient the species of *Aspergillus* could not be established, and in another one, we identified both *Aspergillus flavus* and *Aspergillus niger.*

Once the diagnosis of PCA is confirmed, the treatment must be started as soon as possible. For the systemic treatment of PCA, the main options are polyenes (amphotericin B; liposomal amphotericin-B), azoles (voriconazole, posaconazole, or itraconazole), and Echinocandins (caspofungin; micafungin) [27,28]. The opinions regarding the first-line treatment for aspergillosis are divided between amphotericin B [7,11,19,29] and voriconazole [27,30,31]. The 2021 consensus guidelines for the diagnosis and management of invasive aspergillosis recommend voriconazole with therapeutic drug monitoring as the first-line therapy [32]. The increasing resistance of *Aspergillus* sp. strains to traditional antifungals necessitates antifungal susceptibility testing in order to choose the most appropriate systemic and local treatment from the drugs available in the health facilities where these patients are hospitalized [33,34,35,36].

In our cases, antifungal susceptibility testing was possible only for two of the enrolled patients, and it showed the resistance of two isolates to amphotericin B. All patients had prophylactic antifungal treatment for *Candida* at the moment of PCA diagnosis, which was changed to voriconazole.

Regarding the local treatment for the first case diagnosed with PCA at our clinic, we found ourselves in a difficult situation because we had never encountered such a complication in the evolution of burns until then. Articles on the local treatment of PCA are few and promote the excisional debridement of infected tissues and even amputation in addition to systemic therapy [12,24], and none of them describe the association of this fungal infection with the Meek micrografting technique. This coverage option implies leaving the small square micrografts covered for at least 10 days [6] or even longer with polyamide gauze, which helps keep them in place until graft take occurs and the skin islands begin to merge. During this time, we need to maintain a moist environment, and we cannot visually inspect the micrografts directly.

Taking into account the fact that the first patient had 90% of the TBSA excised and covered with micrografts, an aggressive surgical approach did not seem feasible to us in this case. We decided to remove the polyamide gauze from 30% of the TBSA, and topically, we used voriconazole solution and 1% silver sulfadiazine cream, alternatively. The voriconazole solution was prepared by diluting 400 mg of voriconazole in 500 mL of normal saline. The dressings were changed daily. *Aspergillus* colonies from regions where the polyamide gauze was not removed disappeared, and the micrograft grew and merged. The areas not covered with polyamide gauze developed granulation tissue and required additional skin grafting. Based on this experience, we no longer removed the polyamide gauze in subsequent cases. Instead, we continued with the local alternating application of voriconazole and silver sulfadiazine.

Voriconazole is a second-generation triazole derivative that works by inhibiting fungal sterol-14-alpha-demethylase, a cytochrome-dependent enzyme associated with ergosterol synthesis, thereby impairing cell membrane formation [17,37]. There are many published works on the safety and efficacy of voriconazole as a systemic treatment for invasive aspergillosis, but there is little information about the topical use of antifungal agents [37,38,39].

As far as we know, there is only one case report that describes the use of voriconazole as a topical treatment for a 19-year-old female who had a bone marrow transplant and developed a 5 × 5 cm non-healing skin lesion at the site of a previous insulin injection that was attributed to *A. flavus* complex [26]. For systemic treatment, amphotericin B and caspofungin were used. After the excision of the lesion and repeated surgical debridement, since the evolution was unfavorable, it was decided to apply a local solution of 1% voriconazole twice a day, which led to the diminishing of the wound to 2.5 × 3 cm in 5 weeks.

Fournier et al. reported the case of a 33-year-old male admitted with 92%-TBSA burns who developed PCA that was successfully managed with systemic voriconazole and topical terbinafine cream [15]. The utilization of amphotericin B both systemically and topically was described by Aries for a 53-year-old woman hospitalized for 60%-TBSA thermal burns complicated with *Aspergillus tamarii* cutaneous infection [12]. It is important to highlight the need for both systemic and local antifungal treatment since local treatment alone may lead to ineffective infection control and further risk of transmission [40].

Successful clinical and mycological healing was achieved in five of six cases as a result of the systemic and topical antifungal treatment. In one case, the patient died shortly after being diagnosed with PCA because of the severity of the initial burns, with significant airway involvement, leading to multiple system organ failure. Our experience proves that, although the features of Meek micrografting may create a convenient environment for fungal growth, thorough local care keeps the infection from spreading. At the same time, we found that PCA does not compromise graft take or the patient’s general condition, at least in our teenage patient sample.

Although not observed in our study, systemic complications from fungal dissemination may occur in severely burned patients, resulting in a poor prognosis and increased mortality [41,42]. Severe burns initiate a complex cascade of events, involving immune and inflammatory responses, metabolic changes, and burn-induced coagulopathy. Despite advances in burn treatment, the complex nature of burn trauma continues to present challenges in preventing and managing both systemic and wound-specific complications [43].

The increased mortality associated with invasive fungal infections in burn patients requires prompt efforts for early diagnosis and treatment [41,42]. Systemic manifestations can occur as a consequence of fungal infection development in immunocompromised patients, often requiring prolonged hospitalizations. These manifestations may include respiratory, digestive, and hematologic complications, potentially progressing to multiple organ failure and death [17,44,45,46,47,48].

Invasive pulmonary aspergillosis commonly affects immunocompromised individuals. *A. fumigatus* complex is the primary pathogen, followed by *A. flavus* complex, *A. niger* complex, and *A. terreus* complex. *Aspergillus* infection in burn-related inhalation injuries presents with atypical symptoms, making diagnosis challenging and treatment prone to errors. Because such cases are rarely dealt with, early diagnosis and targeted anti-*Aspergillus* therapy are crucial for improving patient survival [49].

## 5. Conclusions

PCA is a rare invasive fungal infection that may complicate the evolution of extensively burned patients. The use of new surgical techniques, like Meek micrografting, to cover cutaneous defects resulting from burn eschar excision represents an additional risk for the occurrence of PCA and its treatment due to the polyamide gauze that covers the micrografts. When using this technique on extensive burns, careful monitoring for PCA should be carried out on a regular basis. Our study demonstrated that the early removal of the polyamide gauze is not mandatory since early detection and appropriate topical antifungal agents combined with systemic treatment may save the infected grafts and limit the spread of the infection. Thus, some aggressive local measures, such as surgical excision until reaching unaffected viable tissues or segment amputation, can be avoided. This experience may help orient burn teams on how to deal with such complex clinical cases. To define the best diagnostic steps and establish a management protocol, there is a need for further studies, with larger samples of patients.

## Data Availability

The original contributions presented in this study are included in the article; further inquiries can be directed to the corresponding authors. The data can be obtained from the corresponding author upon personal request.

## Abbreviations

The following abbreviations are used in this manuscript:

PCA	Primary cutaneous aspergillosis
TBSA	Total body surface area
ABSI	Abbreviated Burn Severity Index
MIC	Minimum inhibitory concentration
